# Synthesis of a Pseudo-Disaccharide Library and Its Application to the Characterisation of the Heparanase Catalytic Site

**DOI:** 10.1371/journal.pone.0082111

**Published:** 2013-11-18

**Authors:** Victoria Vinader, Mohamed H. Haji-Abdullahi, L. H. Patterson, Kamyar Afarinkia

**Affiliations:** Institute of Cancer Therapeutics, University of Bradford, Bradford, United Kingdom; Taipei Medical University, Taiwan

## Abstract

A novel methodology is described for the efficient and divergent synthesis of pseudodisaccharides, molecules comprising of amino carbasugar analogues linked to natural sugars. The methodology is general and enables the introduction of diversity both at the carbasugar and the natural sugar components of the pseudodisaccharides. Using this approach, a series of pseudodisaccharides are synthesised that mimic the repeating backbone unit of heparan sulfate, and are tested for inhibition of heparanase, a disease-relevant enzyme that hydrolyses heparan sulfate. A new homology model of human heparanase is described based on a family 79 β-glucuronidase. This model is used to postulate a computational rationale for the observed activity of the different pseudodisaccharides and provide valuable information that informs the design of potential inhibitors of this enzyme.

## Introduction

**Figure 1:**
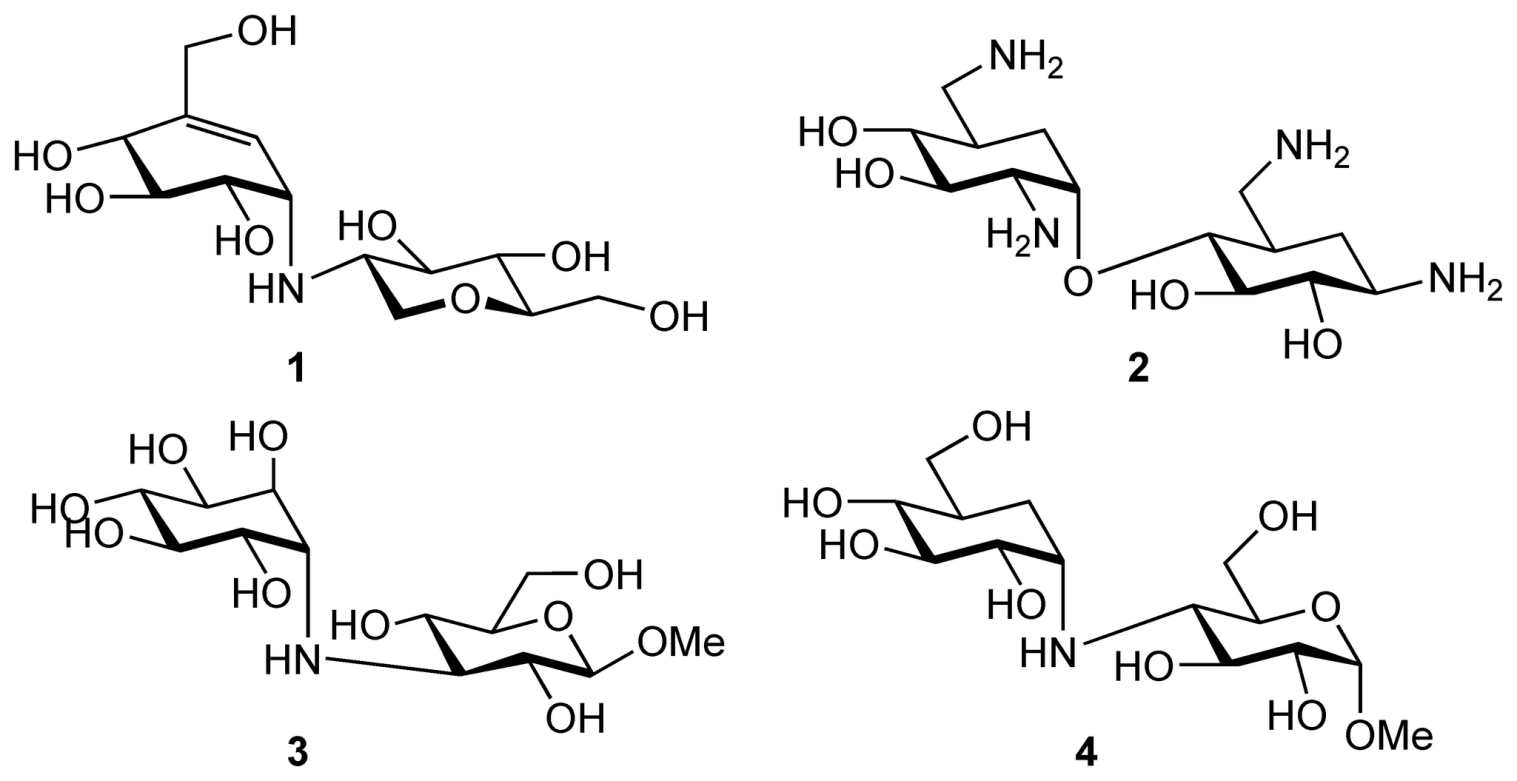
A selection of biologically active pseudodisaccharides.

Glycosyl hydrolases control many significant biological transformations, and are implicated in numerous pathophysiological events[1,2,3]. Therefore, chemical agents that can modulate the activity of these enzymes are of great value, both as biological tools for understanding disease mechanisms, and as potential therapeutic agents[4,5]. One of the most potent and selective classes of small molecule glycosyl hydrolase inhibitors are pseudodisaccharides, molecules comprising of a natural saccharide linked to a pseudomonosaccharide. Examples of pseudodisaccharides with activity against glycosyl hydrolase include natural products salbostatin, **1**[6] and neamine, **2**[7] as well as synthetic α-glucosidase inhibitors **3**[8] and **4**[9] (Figure 1). The use of pseudodisaccharides as glycosyl hydrolase inhibitors is potentially more advantageous than the use of pseudomonosaccharides, for example carbasugars[10,11,12] and azasugars[13,14], because they can achieve greater potency and selectivity [15]. This is postulated to be due to the enhanced binding affinity of pseudodisaccharides as the result of the increase in enzyme-substrate interactions, which leads to a better competitiveness with the enzyme’s natural substrate within the active site.

Access to libraries of pseudodisaccharides for biological evaluation is an important step towards developing a glycomic approach to the identification of both biological probes and drug discovery hits that target glycosyl hydrolases. Pseudodisaccharide libraries can be employed not only to identify new, more potent inhibitors, but also used to probe the catalytic site of an enzyme, to gain a better understanding of its mode of action. However, despite the significance of pseudodisaccharide libraries, there are no general methodologies applicable to their preparation reported so far.

**Figure 2:**
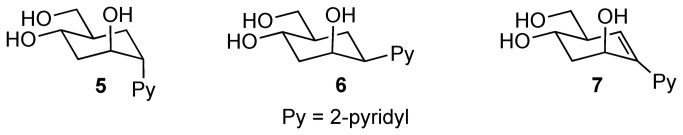
A previously prepared focused library to probe the glycosyl hydrolase enzyme binding pocket.

Our group has pioneered the application of Diels-Alder cycloadditions[16,17,18,19] to the synthesis of pseudomonosaccharides (carbasugars[20] and azasugars[21]), pseudodisaccharides[22,23], and other complex organic molecules[24]. Recently, we have applied this methodology to an efficient and divergent synthesis of a set of pseudomonosaccharides **5**, **6** and **7** (Figure 2), to explore the role of a basic group at the pseudoanomeric position of glycosyl hydrolase enzymes, and demonstrated the usefulness of these molecules in probing the enzyme binding pocket at the anomeric position of mannosidase enzymes[25].

**Figure 3:**
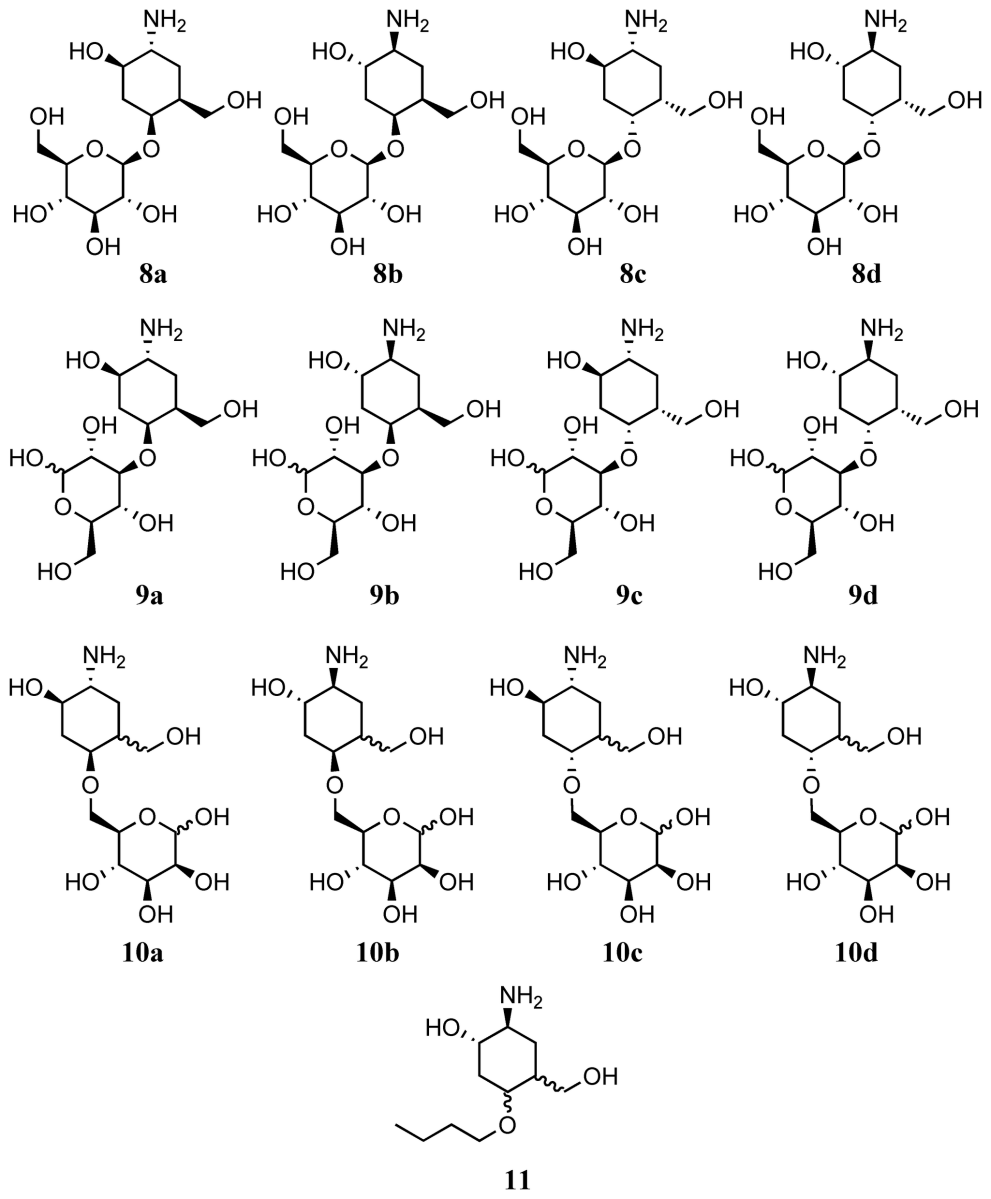
Molecules in the pseudosaccahride libraray, 8a-8d, 9a-9d and 10a-10d, and compound 11.

**Figure 4:**
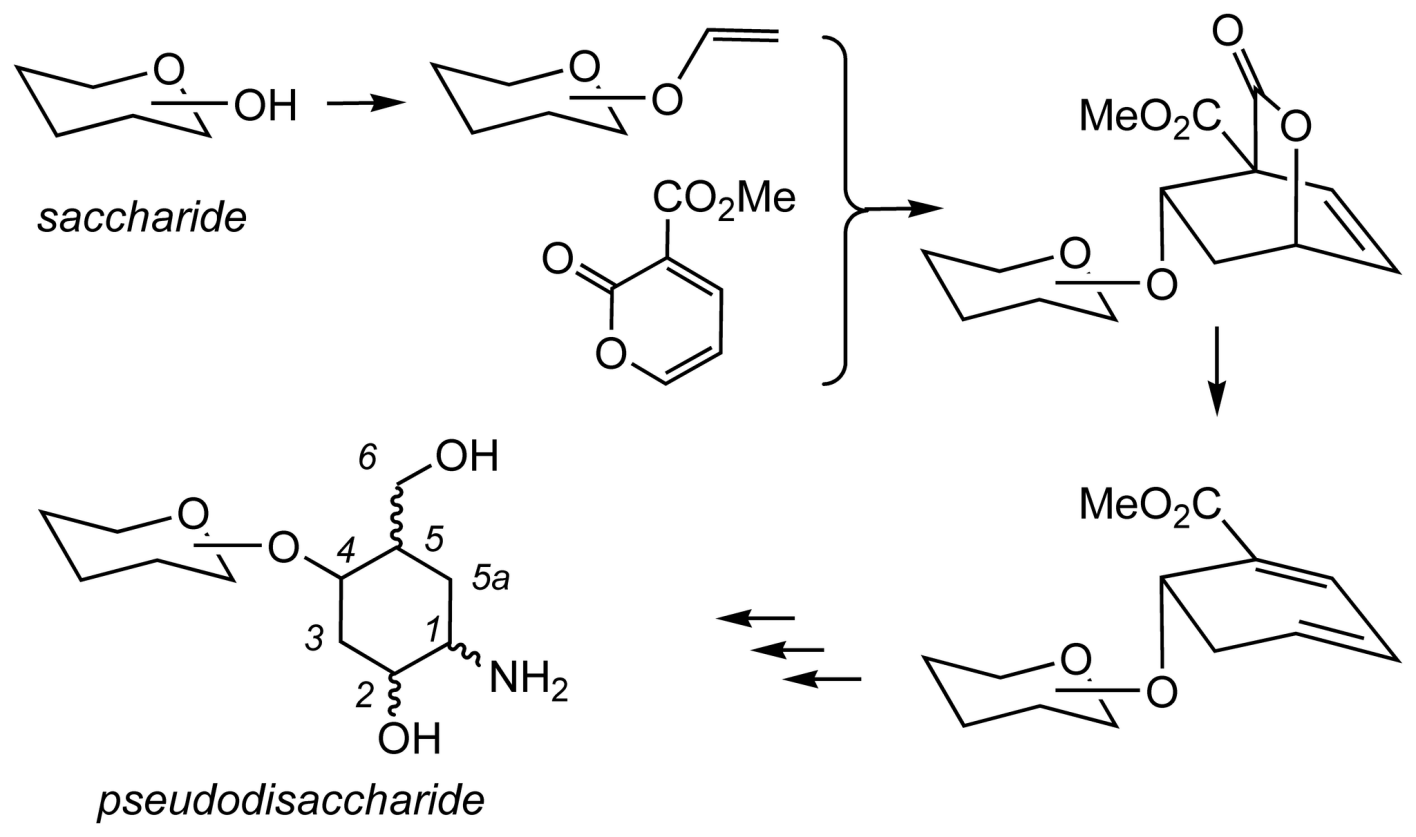
A proposed diversity oriented route to pseudodisaccharides.

In continuation of these studies, we now report an extension to our methodology which enables us to report a straight forward and divergent synthesis of a library of pseudodisaccharides **8a**-**8d**, **9a**-**9d** and **10a**-**10d** (Figure 3) comprising a natural sugar linked to an aminocarbasugar, according to the general route shown above (Figure 4). This approach starts from any given natural sugar with an unprotected hydroxyl group. The free hydroxyl group is first converted to a vinyl ether, and this vinyl ether is then used to construct a carbasugar unit. Hence, our approach is general, and enables introduction of diversity both at the carbasugar component as well as the natural sugar component of the pseudodisaccharides. Furthermore, we showcase the significance of the such libraries by using the synthesized molecules to probe the binding site of a disease-significant glycosyl hydrolase, heparanase, and show the advantage of pseudodisaccharides **8a**-**8d** compared with analogous pseudomonsaccharide, **11** (Figure 3) in these studies.

## Results and Discussions

### Preparation of pseudodisaccharides library and pseudomonosaccharide, 11

**Figure 5:**
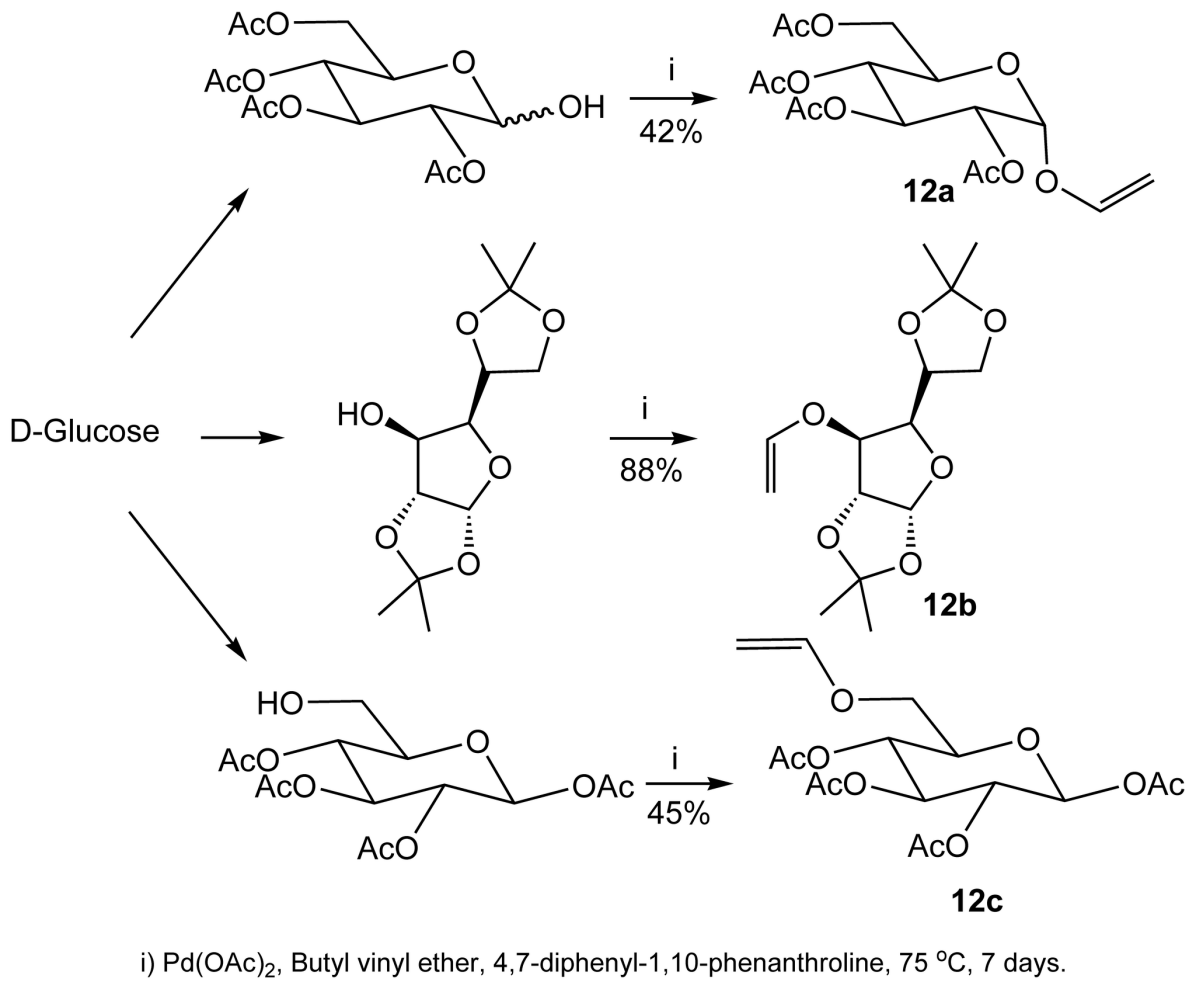
Preparation of vinylsugars 12a, 12b and 12c.

**Figure 6:**
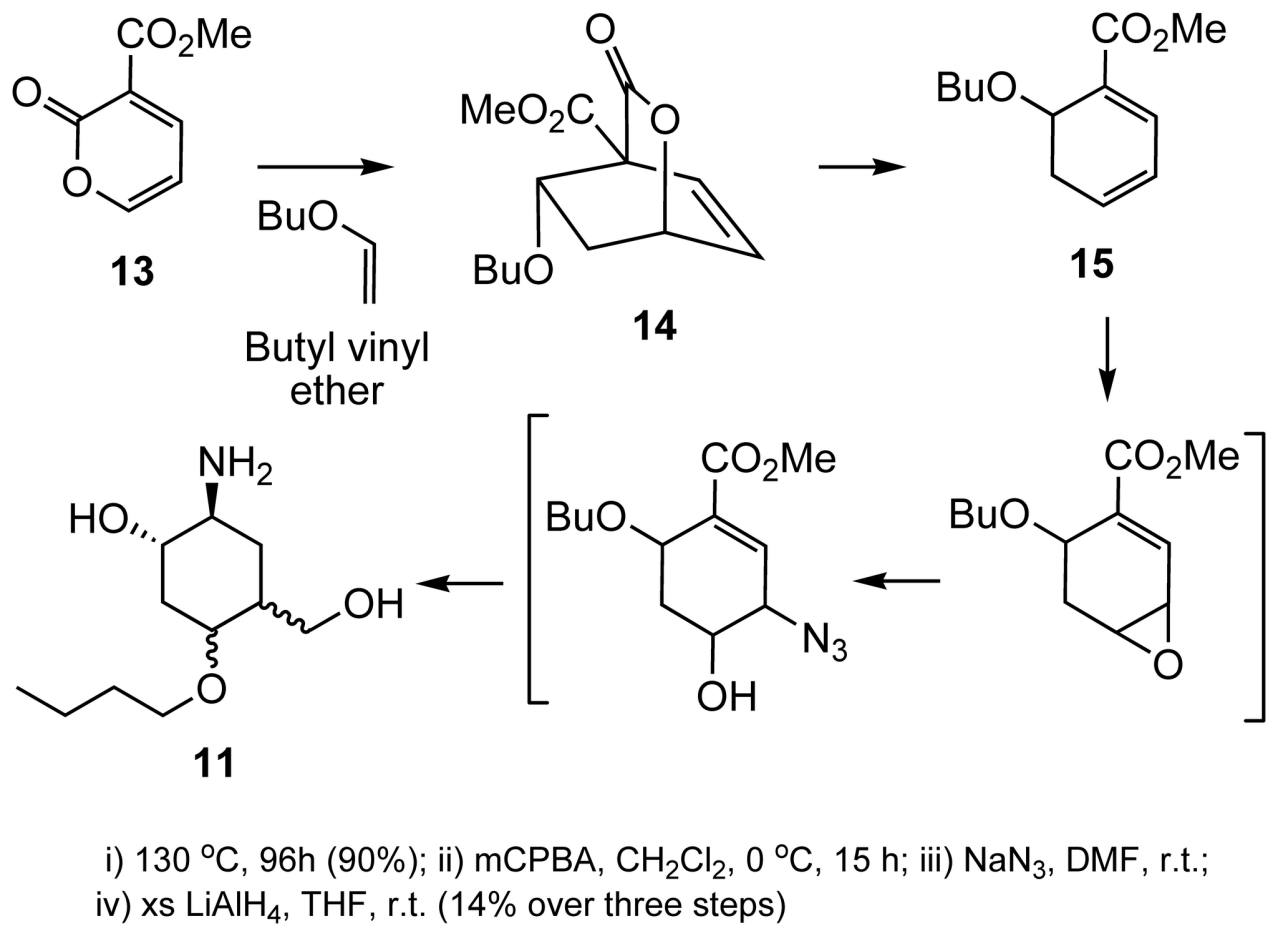
Preparation of compound 11.

Starting from glucose, we first prepared vinylsugar **12a-12c** via transetherification with butyl vinyl ether, in the presence of Pd(II) as a catalyst (Figure 5)[26,27]. These vinyl sugars were transformed to the corresponding pseudosaccharides through a multistep chemical synthesis outlined below (Figure 6). Butyl vinyl ether undergoes facile Diels-Alder cycloaddition with pyran-2-one **13**, resulting initially in the formation of cycloadduct **14**, followed by the loss of bridging CO_2_ on prolonged heating to give dihydrobenzene **15**. Loss of the bridging CO_2_ for similar systems has been shown by us[22,23], Markó[28] and Posner[29] to be facile. Compound **13** was transformed to pseudodisaccharide **11**. We observed no selectivity in these transformations and therefore, **11** was obtained as an inseparable mixture of diastereomers (Figure 6). 

**Figure 7:**
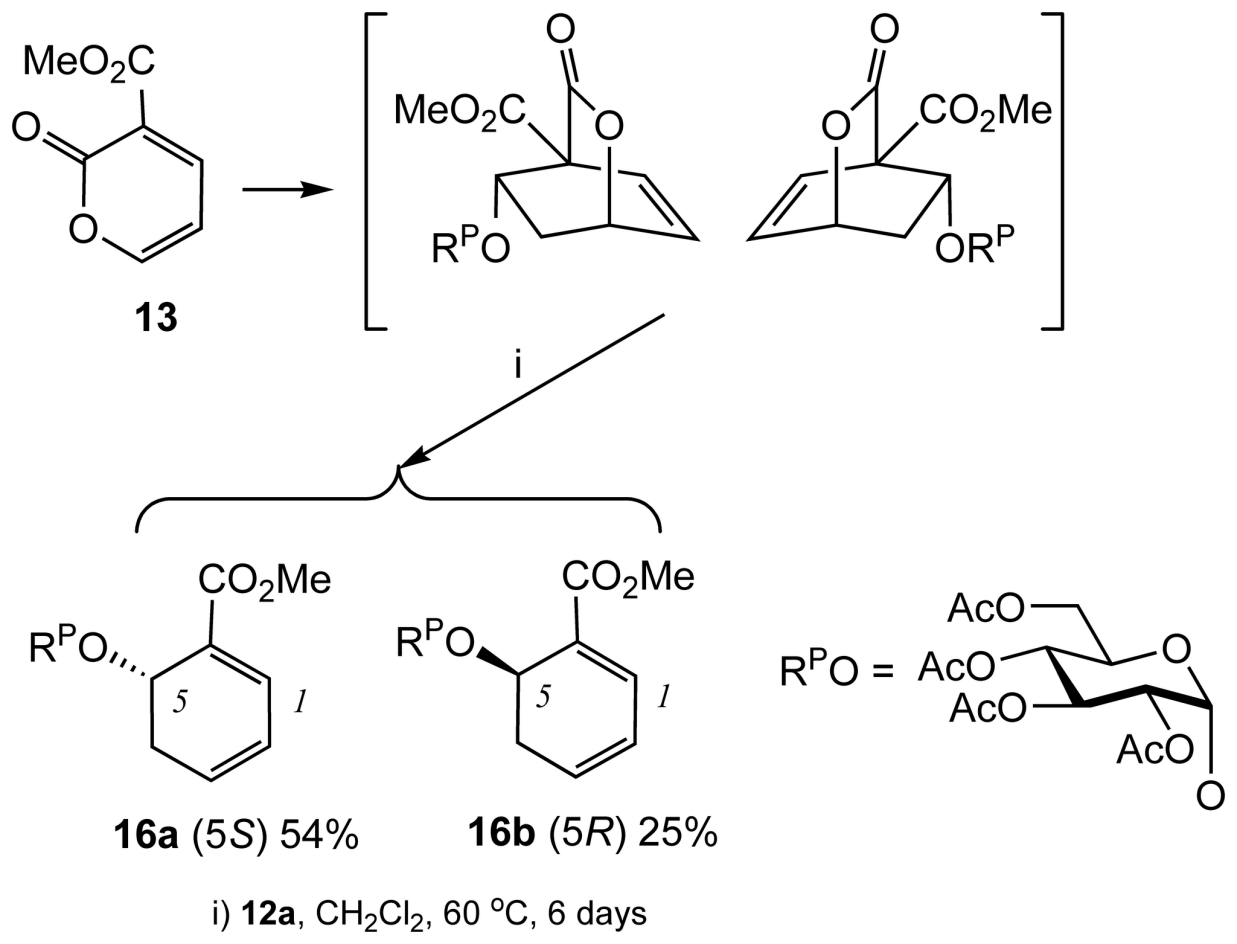
Preparation of dihydrobenzenes 16a and 16b.

**Figure 8:**
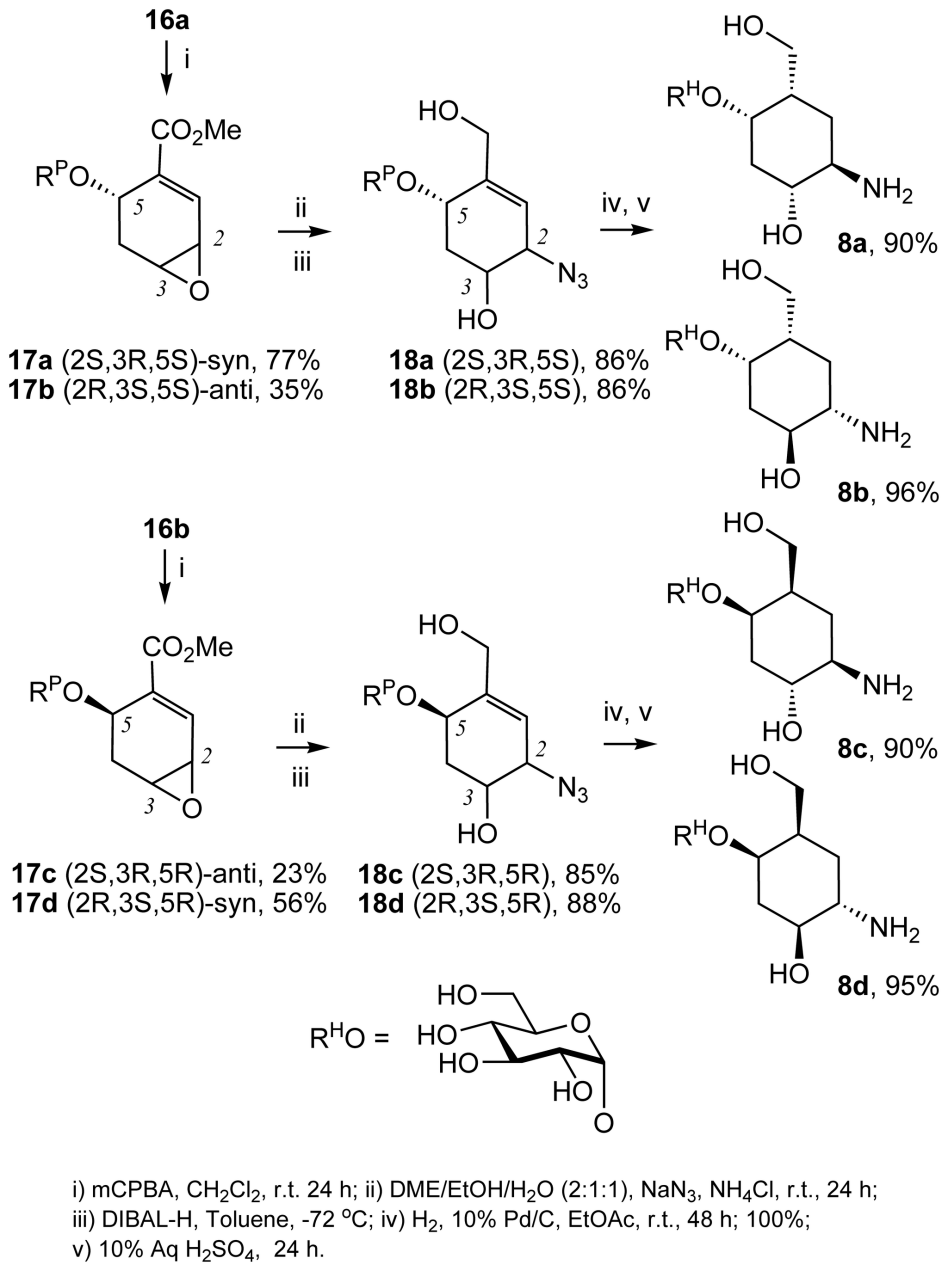
Preparation of compounds 8a-8d, 9a-9d and 10a-10d.

**Figure 9:**
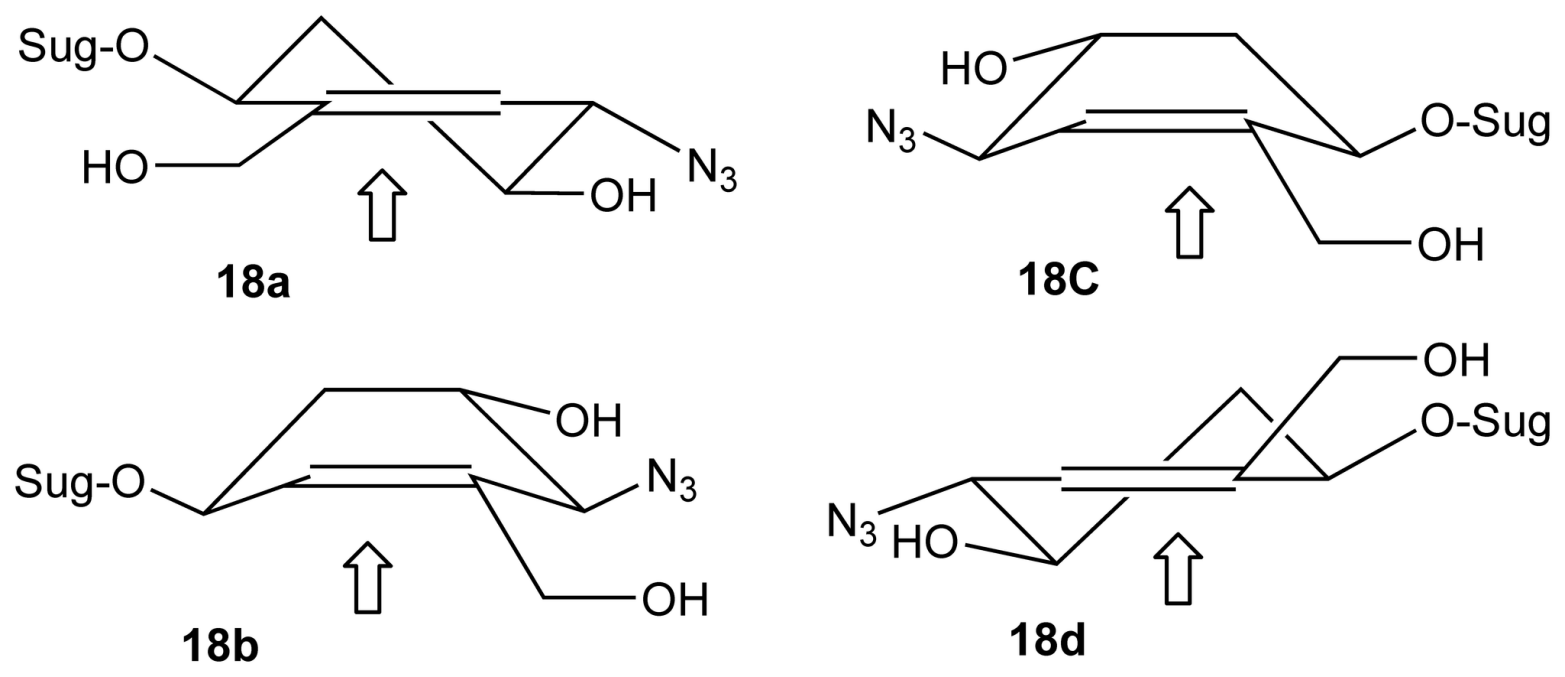
Rationale for the selectivity in the hydrogenation step for 18a-18d. Hydrogen is transferred from the surface of the catalyst to the least hindered face of the alkene shown by an arrow.

In contrast, vinylsugar **12a** underwent the same sequence of reactions but individual stereoisomers could be separated (Figure 7). Diels-Alder cycloaddition between **12a** and **13** was followed by the loss of bridging CO_2_ on prolonged heating to give dihydrobenzene **16a** and **16b**. Following the same sequence of reactions, treatment of each of the dihydrobenzenes with mCPBA afforded the corresponding 2,3-epoxides. For example, **16a** afforded *syn* **17a** and *anti* **17b**, and **16b** afforded *anti* **17c** and *syn* **17d** (Figure 8). The assignment of the configuration of the stereoisomers was based on the correlation of the NMR signals and coupling constants on the X-ray crystallography in the series originating from vinylsugar **12b**[22,23]. Regioselective ring opening of the epoxides with sodium azide followed by a reduction of the ester group afforded **18a**-**18d**. Subsequent concurrent hydrogenation of the alkene and azide functions followed by deprotection of the natural sugar component, afforded pseudodisaccharides **8a**-**8d**. (Figure 8). Hydrogenation of **18a**-**18d** proceeded with stereoselectivity. Presumably, this is because regardless of the configuration of the carbon atoms bearing the hydroxyl and amine functions, the molecule adopts a twisted conformation resulting in the introduction of the hydrogen against the face containing the bulky O-Sug substituent (Figure 9). Finally, the synthesis of the library was completed by carrying out the same sequence of reactions on vinylsugars **12b** and **12c**, affording pseudodisaccharides **9a**-**9d** and **10a-10d** respectively (Figure 3). Interestingly, the hydrogenation step in the sequence leading to **10a-10d** was not stereoselective, presumably because of the smaller steric bulk by the OSug group in this series (see File S1 in supporting information for all preparative methods and spectroscopic characterisation).

### Biological evaluation of the pseudodisaccharide libraries

Heparanase is a family 79 glycosyl hydrolase (*endo*-β-D-glucuronidase) which exclusively degrades heparan sulfate (HS), a cell surface proteoglycan which plays a significant role in the biology of the cell[30,31]. HS interacts with a multitude of extracellular matrix (ECM) constituents, growth factors and proteins through its polysaccharide component[32]. These interactions regulate a wide variety of biological activities of the cell, and thus control a range of (patho)physiological processes, including cellular development, angiogenesis, blood coagulation, viral recognition and, tumour dissemination. Degradation of HS by the elevated levels of heparanase during disease, alters these interactions. Hence, heparanase promotes the increase in tumour neovascularisation and degradation of the extracellular matrix thus modulating tumour cell-matrix adhesion and promoting migration. The evidence for heparanase in tumour growth and metastasis of many cancers including breast[33,34], myeloma[35]and bladder[36] is well documented. 

**Figure 10:**
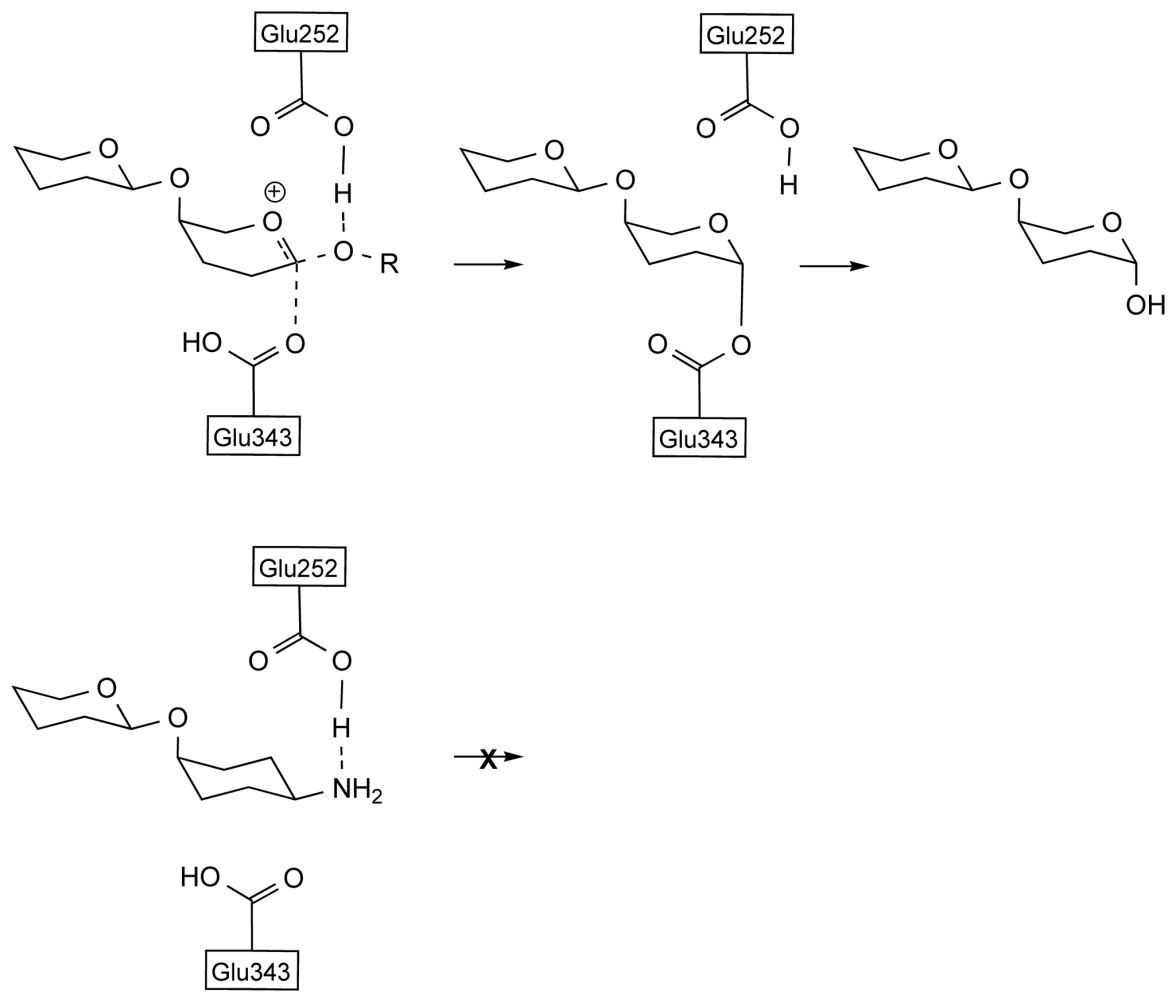
Mechanism of the hydrolysis by heparanase. (a) Role of Glu252 and Glu343 residues at the catalytic site (b) The interaction between the of amino substituent at the pseudoanomeric position and carboxyl residues.

Therefore, identification of potent and selective inhibitors of heparanase is a significant step forward in combating disease. In this context, mechanism based rational design of molecules that can competitively bind at the catalytic site of heparanase has been an important goal in medicinal chemistry. These efforts are informed by the mechanistic knowledge of the mode of action of heparanase. Heparan sulfate, the natural substrate for heparanase, is a variably sulfated, linear, repeating chain of glucosamine and hexuronate (either D-glucuronate or L-iduronate) units. Even though there are other sulfated glycosaminoglycans in the glycocalyx, cleavage of heparan sulfate by heparanase, which occurs at the (1,4)-link between hexuronate and glucosamine, is both efficient and selective. Interestingly however, heparanase, like many other glycosyl hydrolases, appears to have some tolerance to interacting with saccharides other than its natural substrate. For example, short chain, non-sulfated disaccharides like GlcA-GlcNAc are substrates for the enzyme but are not catalytically turned over[37]. Furthermore, heparanase is inhibited by polysaccharides which do not have significant configurational identity to its natural substrate. For instance, heparanase is inhibited by a sulfated pentamannose[38], even though this particular saccharide is not represented in heparan sulfate. This indicates that there is flexibility for saccharides other than the natural substrate within the catalytic domain of this glycosyl hydrolase. Within the catalytic domain of heparanase, in addition to the residues which are responsible for the recognition of the substrate, two acidic residues, Glu225 and Glu343, are identified by site directed mutagenesis studies[39] to be important. It is postulated that Glu225 acts as a proton donor to the exocyclic oxygen of the glycosyl link, to activate it as a leaving group and initiate the cleavage process, whilst Glu343 acts as a nucleophile in the proposed retaining mechanism of the glycolysis (Figure 10). Based on this knowledge, we postulate that molecules with a basic group at or near the anomeric position, provided that basic group can interact strongly with the acidic residues at the active site, can bind more tightly and hence more effectively inhibit the enzyme (Figure 10)[25]. 

**Figure 11:**
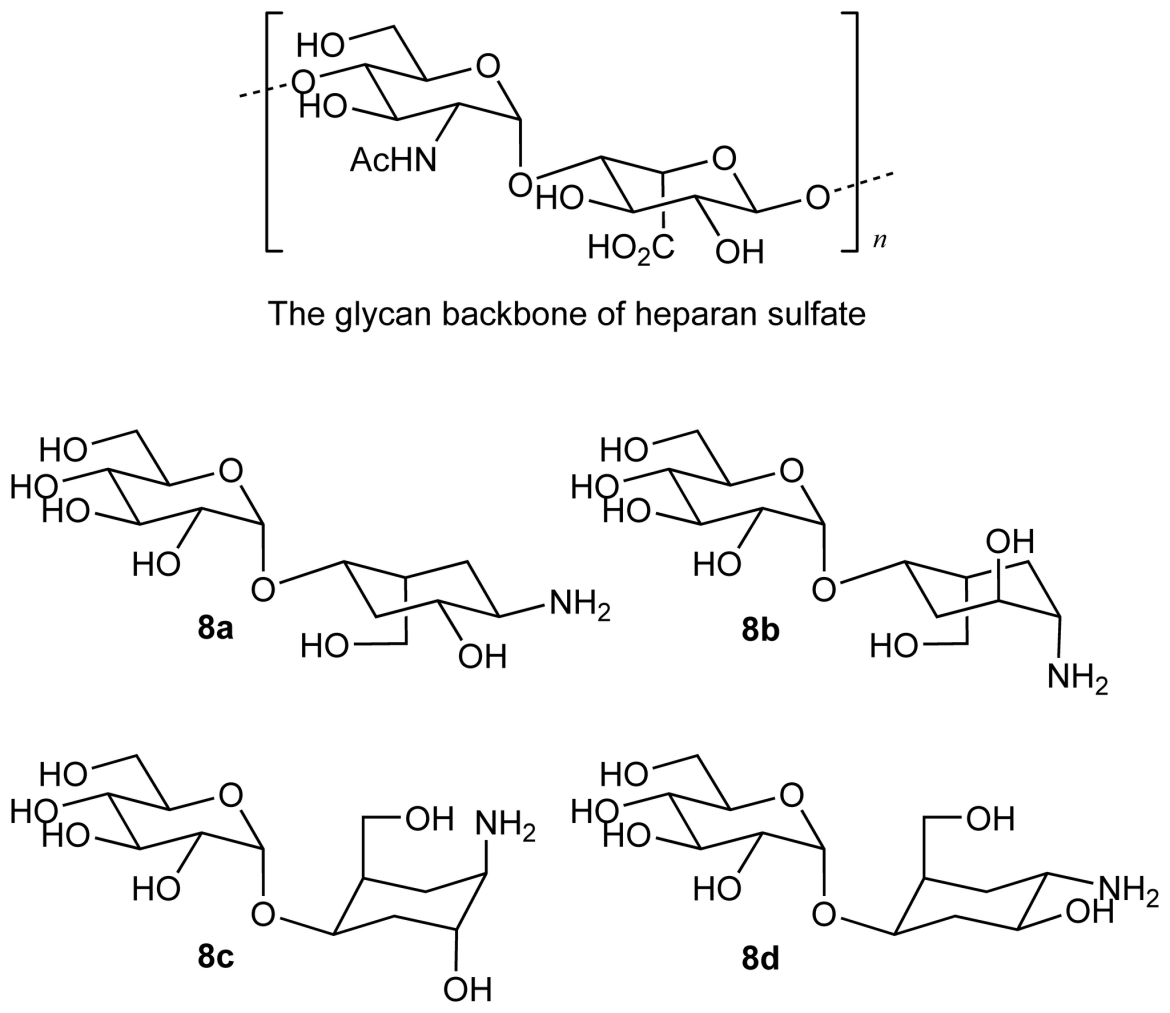
Configuration of compounds screened against heparanase and their relationship to heparan sulfate polysaccharide backbone.

In view of the propensity of heparanase to bind to non-sulfated, non-configurationally similar saccharides[37,38], we considered compounds **8a**-**8d** might bind to heparanase and inhibit its catalytic function. Structurally, compounds **8a**-**8d** can be rationalized to partially mimic the repeating unit of HS (Figure 11). However, the basic amine group and other polar substituents on the carbasugar component in these molecules are configurationally arranged differently. Therefore, we decided to measure inhibition of heparanase by compounds **8a**-**8d** to assess whether the configuration of these amino substituents, influences potency of inhibition. Comparing the activity of this set of compounds should provide information about the flexibility of the enzyme in accommodating different configurations around the pseudosaccharide unit at the catalytic site. We decided to also investigate compound **11** to see whether the postulate that a pseudodisaccharide analogue is a better inhibitor than a pseudomonosaccharide can be demonstrated in this case.

**Figure 12:**
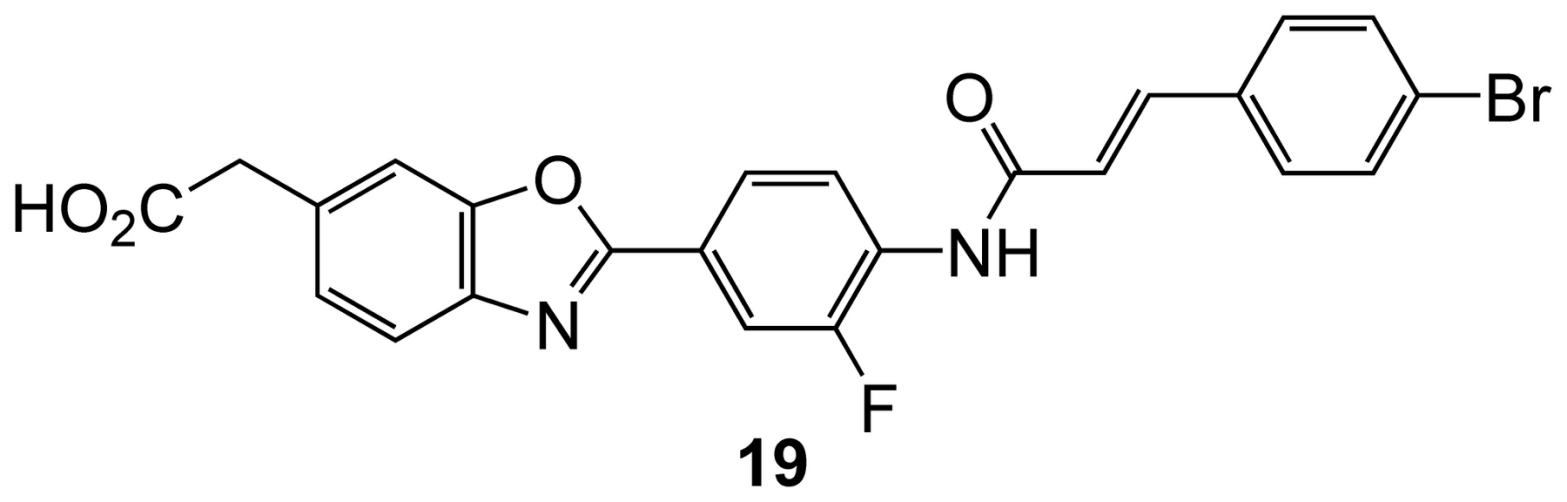
Compound 19.

We tested compounds **8a**-**8d**, and the pseudomonosaccharide **11**, as well as the known heparanase inhibitor, OGT2115, **19** (Figure 12)[40], as a positive control. Different concentrations of the test compounds were incubated with biotinylated heparan sulfate (HS) and human heparanase for a specified time. In the presence of inhibitors, the rate of hydrolysis of HS by heparanase to generate a short chain HS is diminished. Remaining full length HS was detected by binding to immobilised CBD-FGF. CBD-FGF is a fusion protein of cell-binding domain of human fibronectin and human fibroblast growth factor. This binding was quantified by avidin-peroxidase (avidin-POD) and a chromogenic substrate.

In this assay, compound **19**, a known heparanase inhibitor[40], exhibited an IC_50_ for short chain HS formation of 10±2 µM (Table 1). Pseudomonosaccharide **11** showed no inhibition of heparanase whilst pseudodisaccharides **8a**, **8b** and **8d** showed moderate activity with IC_50_’s in 1 mM range. However, no significant activity for **8c** was observed (Table 1, see File S2 in supporting information for dose response curves). The level of heparanase inhibition by compounds **8a**, **8b** and **8d**, although modest, is quite acceptable for designating these molecules as hits and using them as a starting point in a potency optimisation programme. This also demonstrates the usefulness of the pseudodisaccharide libraries for identification of molecules that can interact with glycosylhydrolases such as heparanase. 

**Table 1:** The IC_50_ values of compounds 19 (as reference), 11 and 8a-8d against human heparanase.

**Compound**	**IC_50_ (n=3)**
OGT2115, **20**	10±2 μM
**11**	> 10 mM
**8a**	1.2±0.3 mM
**8b**	0.8±0.2 mM
**8c**	> 10 mM
**8d**	0.8±0.2 mM

The values are mean of three readings ± standard error.

### Molecular modeling successfully rationalize the observed activities

We have previously used modeling studies to successfully explain observed potencies of inhibitors of Golgi mannosidase II, another glycosyl hydrolase[25]. In view of this, we decided to determine the binding energies of pseudodisaccharides **8a**-**8d** inside the heparanase catalytic domain and investigate if a correlation exists between heparanase inhibition by these compounds and their enzyme fit. Since no crystal structure for heparanase exists, we used a homology model for these computational studies. There are at least three homology models of heparanase (two computational[41,42] and one NMR based[43]) described in the literature. However, none of these models is based on a family 79 glycosyl hydrolase, the same family to which heparanase belongs. Very recently, a crystal structure of a family 79 β-glucuronidase from *Acidobacterium capsulatum* (pdb code 3VNY)[44] was reported. This report also includes crystal structures of the enzyme with both glucuronic acid, as a competitive inhibitor (pdb codes 3VNZ)[44] and 2-fluoro-2-deoxyglucose as a suicide inhibitor (pdb code 3VNO)[44] within the active site. Because it belongs to the same hydrolase family as heparanase, we felt that a new homology model based on this crystal structure would provide a better tool for a computational investigation. Indeed, we found there to be good agreement between the sequence of heparanase and the chosen template as well as between the predicted secondary structure of heparanase and the actual secondary structure of the template (see File S3 in supporting information).

**Figure 13:**
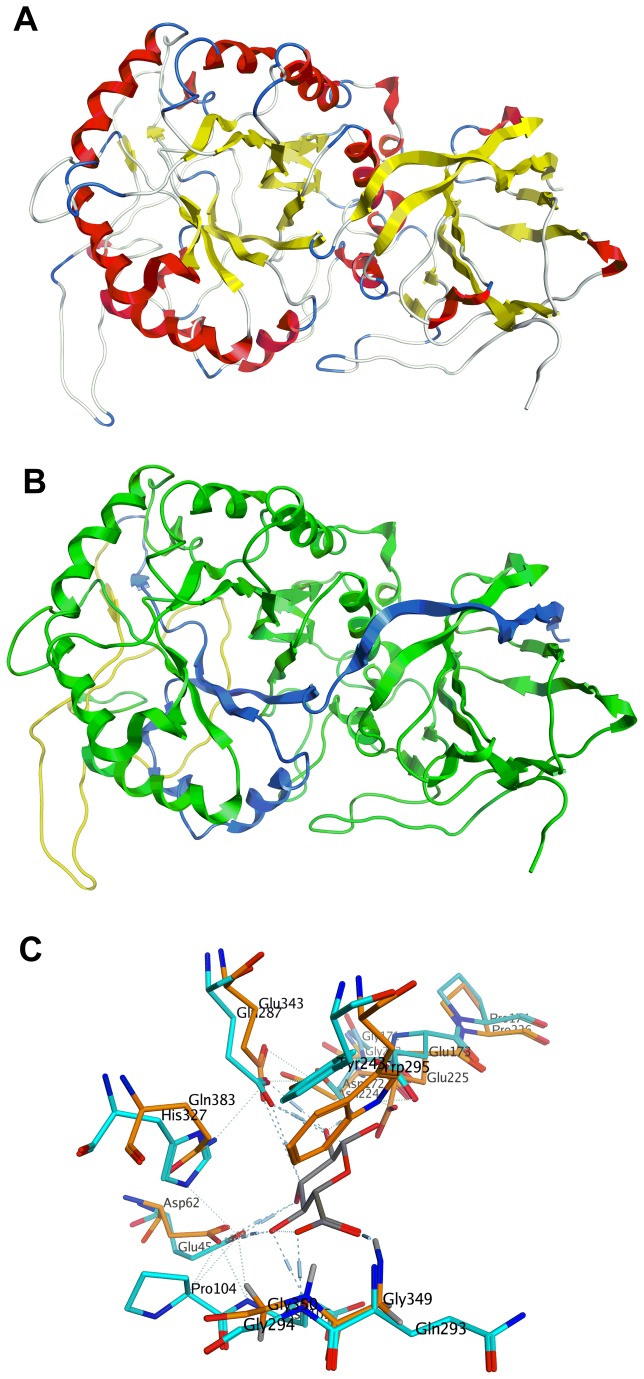
Homology model of heparanase showing: (a) the TIM barrel fold; (b) the 8kD unit (blue), the 50 kD unit (green) and the linker (yellow); (c) a comparison of the active site of the modelled heparanase and the tempelate, 3NVY.

A homology model of the full length heparanase, comprising the 8kD subunit (Gln36-Glu109), the linker unit (Ser110-Gln157), and the 50 kD unit (Lys158-Ile543) which contains the active site, was constructed using Molecular Operating Environment (MOE) software (Chemical Computing Group)[45]. There is good agreement between the sequence of heparanase and the chosen template (see supporting information). The enzyme possesses a TIM barrel fold consistent with other *endo*-β-D-glucuronidases. The catalytic residues Glu225 and Glu343[39], are flanked by the heparin/HS binding regions lined with highly basic residues. The binding pocket for the heparanase model has significant similarities with that of 3VNY (Figure 13). Both Trp295 in the model and Tyr243 in the template provide a hydrogen bonding opportunity near the glycosidic linkage of the hexauronate unit. In addition, basic residue His327 and Asn172 interact with the acidic catalytic residue Glu287 and Glu173 in the template. That role is similar to that played by the interaction of Gln383 and Asn224 with the acidic catalytic residue Glu343 and Glu225 in the model.

There is one noticeable difference between the modelled heparanase and the template. In β-glucuronidase from *Acidobacterium capsulatum*, a pocket lined with residues Tyr292, Gln293 and Gly294 surround the C-6 of the hexauronate and there is an interaction between the basic residue Gln293 and the carboxylic function in the glucuronic acid ligand. However, the corresponding pocket in the modelled heparanase is lined with Tyr348, Gly349, and Gly350. Whilst the lack of a basic residue in this pocket can be significant in the modelled heparanase, we saw no evidence from modelling studies to suggest any significance for this observation.

**Figure 14:**
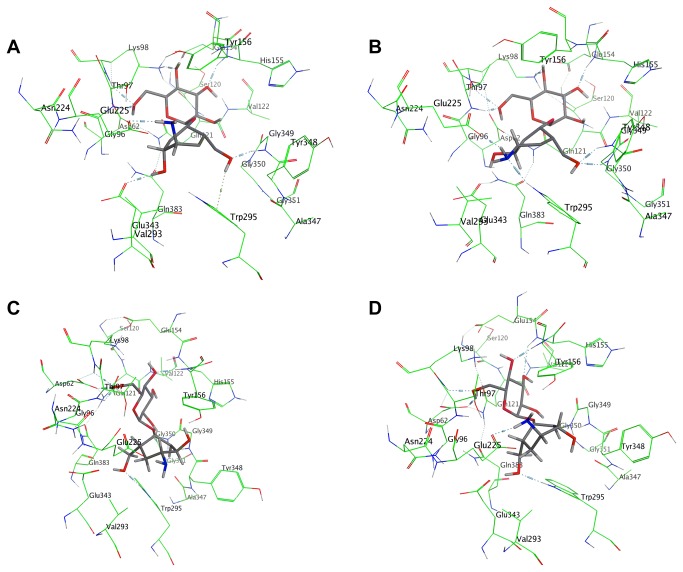
Binding modes of inhibitors within the binding site of heparanase (a) 8a; (b) 8b; (c) 8c; and (d) 8d.

Energetically accessible conformations of **8a**-**8d** and **11** were docked inside the enzyme active pocket, visually inspected for correct orientation and placement near catalytic residues, and energy minimised (Figure 14). All molecules adopted a boat conformation in the active site which is in agreement with previous observations[25,46]. 

Compound **11** exhibited a relatively weak binding affinity (-5.46 Kcal/mol), which can explain why no significant inhibition of recombinant human heparanase was observed for this compound. This confirms the postulate that a pseudomonosaccharide is a poorer substrate than a pseudodisaccharide for inhibition of a glycosyl hydrolase. Binding affinities for **8a**, **8b**, **8c** and **8d** were computed as -6.72 kcal/mol, -6.48 kcal/mol, -5.98 kcal/mol and -6.43 kcal/mol respectively. 

**Figure 15:**
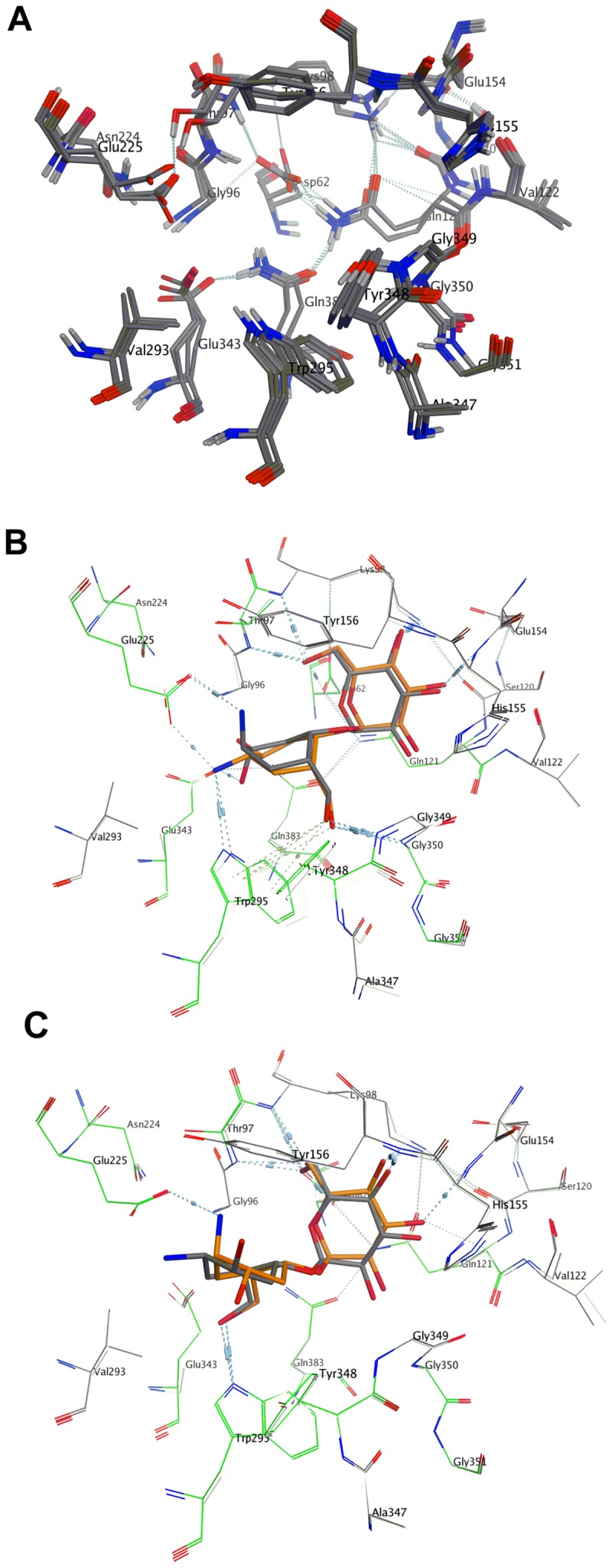
Comparison between the binding modes of 8a-d. (a) Superimposition of the position of residues at binding site of heparanase when it is bounds to inhibitors **8a-8d** Inhibitor molecules are removed for clarity. (b) Superimposition of the inhibitors **8a** (gold), **8b** (grey), at binding site of heparanase shows that despite differences in conformation there is interaction with the amine group in both molecules; (c) Superimposition of the inhibitors **8c** (grey) and **8d** (gold) shows that due to differences in conformation, there is no interaction with the amine group in **8c** molecules.

The calculated higher binding affinities for compounds **8a**, **8b**, and **8d** in the catalytic site of the enzyme correlate with the higher potency of these compounds in inhibition of heparanase. Analysis of the modes of binding of these molecules within the catalytic site of heparanase, reveals that even though the molecules do not have the same configuration at the carbasugar unit, the molecules are able to adopt a conformation that allows an interaction between their respective amino group and the Glu225 residue of the catalytic domain (Figure 15). According to the postulated mechanism of action for heparanase (Figure 11), this residue acts as a proton donor. So we propose that in compounds **8a**, **8b**, and **8d** the interaction between the basic amine group of the pseudodisaccharide and the acidic carboxyl side chain in Glu225, enhances the binding between these pseudodisaccharide and the enzyme, resulting in inhibition. The lower calculated binding affinity for compounds **8c** corresponds to the lack of potency in inhibition of heparanase. Analysis of the mode of binding of **8c** within the catalytic site of heparanase, reveals that there is no interaction between its respective amino group and Glu225 residue, consistent with its lack of heparanase inhibition (Figures 9 and 10). This confirms that conformational flexibility in **8a**, **8b**, and **8d** is the factor that enables interaction between the basic amine group of the pseudodisaccharide and the acidic carboxyl side chain in Glu225, thus contributing to their higher affinity, and potency. 

Based on these observations, we can conclude that the basic group at the pseudoanomeric position plays a critical role in the inhibition of heparanase through interaction with Glu225 residue at the catalytic domain. Furthermore, we conclude that there is considerable flexibility within the enzyme binding pocket of heparanase. Hence, those pseudodisaccharide that do not exactly mimic the substrate, can still adopt a suitable conformation to enable them to bind within the heparanase catalytic domain with sufficient affinity to inhibit heparanase. Furthermore, the molecular model of heparanase we constructed provides a practical tool to calculate the binding affinity energies of pseudodisaccharides enabling prediction of their potential as heparanase inhibitors. 

## Conclusion

We have reported a novel approach to discovering molecules that bind and inhibit glycosylhydrolases such as heparanase, by combining “rational design” and screening of diversity oriented libraries. To achieve this, we have developed a new and facile approach to the preparation of a pseudodisaccharide library in which diversity can be introduced both in the carbasugar as well as natural sugar components. In addition, we have used a combination of computational and experimental result for compounds **8a**-**8d** to gain valuable information about the catalytic pocket of a glycosyl hydrolase. Specifically, we conclude that there is a degree of flexibility in the binding pocket of heparanase that can accommodate some configurational changes to the pseudosaccahride component of compounds **8a**, **8b** and **8d**. In view of this, we now plan to carry out similar investigations on **9a**-**9d** and **10a**-**10d** to assess how the configurational changes in the natural sugar component can influence potency of the inhibition, and will report our results in due course.

## Supporting Information

File S1 **Preparation and spectroscopic characterization of exemplar compounds.**
(PDF)

File S2 **Dose-response curves for heparanase inhibition by compounds 8a-d and 19.**
(TIF)

File S3 **Alignment of heparanase (sequence from Uniprot accession number Q9Y251) and β-glucuronidase from *Acidobacterium capsulatum* strain ATCC 51196 (sequence from Uniprot accession number C1F2K5).** Predicted folds are shown (red is an α-helix, yellow is β-sheet and blue is a turn).
(TIF)
