# Supplementary material for: Synthesis of a Pseudo-Disaccharide Library and Its Application to the Characterisation of the Heparanase Catalytic Site
Source: PLoS One. 2013 Nov 18;8(11):e82111. doi: 10.1371/journal.pone.0082111 (PMC3832595; doi:10.1371/journal.pone.0082111)
Supplement: File S1 — Preparation and spectroscopic characterization of exemplar compounds. (PDF) [file pone.0082111.s001.pdf]

Supporting information

Synthesis of a Pseudo-Disaccharide Library and its Application to the Characterisation of the Heparanase Catalytic Site

M. Victoria Vinader, Mohamed H. Haji-Abdullahi, L. H. Patterson and Kamyar Afarinkia

Institute of Cancer Therapeutics, University of Bradford, Bradford, BD7 1DP, United Kingdom.

Tel: (+44) 1274235831
Fax: (+44) 1274233234
E-mail: k.afarinkia@bradford.ac.uk.

Experimental procedure for the preparation of compounds

Compound 8a Pd/C (10 mg) was added to a solution of compound **18a** (155 mg, 0.30 mmol) in EtOAc (10mL) and the mixture was maintained under a hydrogen atmosphere for 24 hours. The catalyst was removed by filtration and the residue was stripped of solvent under reduced pressure. A chilled solution of ammonia in methanol (2M, 5 mL, 33 eq.) was added to the residue, and the resulting solution was transferred to a rotavap and stripped of all volatiles whilst maintaining the solution at 0 °C. The residue chromatographed (2% MeOH in DCM) on a short pad of silica to afford **8a** as a foam (97 mg, 90%). Compounds **8b**, **8c** and **8d** were prepared similarly. IR: (thin film) 3468, 2951, 2111, 2840, 1647, 1450, 1109, 1019 cm^{-1}.^{1}H NMR (D_2O, 600 MHz): δ 1.35-1.41 (m, 1H, H-5a), 1.54 (dt, 1H, *J* = 12.9 Hz, H-3), 2.05 (ddd, 1H, *J* = 3.4, 6.7, 13.9 Hz, H-5a), 2.17 (ddd, 1H, *J* = 3.3, 7.7, 12.9 Hz, H-3), 2.29-2.31 (m, 1H, H-5), 3.02-3.07 and 3.20-3.40 and 3.45-3.62 (7H, overlapping m, H-1, H-2, H-4, H-2', H-3', H-4' and H-5'), 3.66-3.92 (4H, overlapping m, H-6 and H-6'), 5.06 (d, partial 1H, *J* = 3.6 Hz, H-1'). ^{13}C NMR (D_2O, 150.9 MHz): δ 26.29 (C-5a), 34.82 (C-3), 38.32 (C-5), 46.90 (C-1), 59.0 (C-6'), 60.7 (C-6), 68.85 (C-2'), 71.02 (α-anomer C-4'), 74.7 (C-5'), 78.47 (C-4), 81.23 (C-3'), 92.27 (C-1'). LRMS (ESI^+): 324.2 $[M+H]^+$. HRMS (ESI) calcd for $C_{13}H_{26}NO_8$ $[M+H]^+$: 324.1658; Found: 324.1670.

2-amino-5-butoxy-4-(hydroxymethyl)cyclohexan-1-ol, 11. mCPBA (65% w/w, 200 mg, 0.72 mmol) was added to a solution of methyl 6-butoxycyclohexa-1,3-dienecarboxylate, **15** (150 mg, 0.72 mmol) in DCM (5 mL) maintained at 0 °C. After 15h, more DCM (20 mL) was added and the mixture was washed with sat $NaHCO_3$ (3 x 10 mL). The organic extracts were dried over $MgSO_4$ and stripped of solvent under reduced pressure. The residue was taken up in DMF (5 mL) containing NaN_3 (47 mg, 10 eq). After 24 h, DMF was removed first under a stream of nitrogen and then at high vaccum. The resulting foam was dissolved was taken up in EtOAc, and filtered to remove any solid. Pd/C (10 mg) was added to the EtOAc solution and the mixture was maintained under a hydrogen atmosphere for 2 days. Filteration, removal of solvent under reduced pressure and chromatography on the residue (0.5-1% MeOH in CH_2Cl_2) afforded compound **11** (22 mg, 14%) as a diasteromeric mixture. δ 0.94 (t, 3H, *J* = 7.56 H-4'), 1.30-1.49 (m, 4H, H-2' and H-3'), 1.65 (2H, m, H-5a), 1.84 (m 3H, H-4 and H-5), 2.48 (m, 1H, H-1), 3.29-3.43 (m, 3H, H-1' and H-3), 3.45-3.78 (2H, m, H-6); ^{13}C NMR ($CDCl_3$): δ 13.95 & 13.93 (C-4'), 19.36 & 19.38 & 19.40 (C-3'), 30.24-35.0 (C-2', C-5a and C-1), 39.0 & 39.1 & 39.3 &39.5 (C-5), 62.1& 62.3 & 62.7 & 62.8 (C-6), 62.07-63.5 (C-3 and C-2), 69.1& 69.2 & 69.7 (C-1'), 75.1-77.3 (C-4); LRMS (ES^+): 201 $[M+H-OH]^+$; HRMS (ESI) calcd for $C_{11}H_{23}NO_2$ $[M+H-OH]^+$: 201.1729; Found: 201.1730.

Vinyl Sugars **12a**, **12b**, and **12**c were prepared according to literature procedures. Compound **13** is commercially available (SigmaAldrich, Gillingham, UK, cat number 359475).

5-*endo*-Butoxy-3-oxo-2-oxa-bicyclo[2.2.2]oct-8-ene-4-carboxylic acid methyl ester, 14. A solution of 3-carbomethoxy-2(H)-pyran-2-one **13** (537 mg, 3.48 mmol) and butyl vinyl ether (5 mL) was heated for 44 h at 60 °C in a sealed tube. The reaction mixture was cooled to room temperature and evaporated under reduced pressure. The crude product was purified by flash chromatography on silica gel (pre-washed with petrol/EtOAc/Et_3N (92:6:2), eluting with petroleum ether/EtOAc (5:1) to give the compound **14** (680 mg, 77%) as a pale yellow oil. IR: (thin film) 2957, 2925, 2868, 1755, 1739, 1438, 1351, 1280, 1095, 975 cm^{-1}; ^{1}H NMR ($CDCl_3$, 600 MHz): δ 0.84 (t, 3H, *J* = 7.6 H-4'), 1.24 (sextet, 2H, *J* = 7.6 Hz, H-3'), 1.37-1.45 (m, 2H, H-2'), 1.65 (dt, 1H, *J* = 1.72, 13.8 Hz, H-6), 2.56 (ddd, 1H, *J* = 3.8, 7.6, 13.8 Hz, H-6), 3.29-3.33 (m, H, H-1'), 3.42-3.46 (m, H, H-1'), 3.88 (s, 3H, OCH_3), 4.33 (dt, 1H, *J* = 7.6, 1.2 Hz, H-5), 5.23 (ddd, 1H, *J* = 1.7, 3.6, 6.9 Hz, H-1), 6.56 (dd, 1H, *J* = 5.2, 7.7 Hz, H-7), 6.76 (dd, 1H, *J* = 0.9, 7.7 Hz, H-8); ^{13}C NMR ($CDCl_3$, 150.9 MHz): δ 13.76 (C-4'), 19.16 (C-3'), 31.62 (C-2'), 35.28 (C-6), 52.94 (OCH_3), 61.48 (C-4), 69.98 (C-1'), 72.79 (C-5), 74.30 (C-1), 129.75 (C-8), 130.57 (C-7), 167.68 (C=O), 168.84 (C=O); LRMS (ESI^+): 272.2 $[M+NH_4]^+$; HRMS (ESI) calcd for $C_{13}H_{22}O_5N$ $[M+NH_4]^+$: 272.1492; Found: 286.1490.

Methyl 6-butoxycyclohexa-1,3-dienecarboxylate, 15. A solution of compound **14** (208 mg, 0.82 mmol) and DCM (2 mL) was heated for 36 h at 100 °C in sealed tube. The reaction mixture was cooled to room temperature and evaporated at reduced pressure. The crude product was purified by flash chromatography on silica gel, eluting with petroleum ether/EtOAc (5:1) to give compound **15** (163 mg, 95%) as a pale yellow oil. Spectroscopic data in agreement with those previously published. ^{1}H NMR ($CDCl_3$): δ 0.84 (t, 3H, *J* = 7.56 H-4'), 1.30 (sextet, 2H, *J* = 7.39 Hz, H-3'), 1.37-1.49 (q, 2H, J = 6.87 Hz, H-2'), 2.37-2.47 (m 1H, H-5), 2.80 (ddd, 1H, *J* = 1.20, 5.50, 19.42 Hz, H-5), 3.29-3.33 (dt, H, J = 6.70, 9.28 Hz, H-1'), 3.50 (dt, H, J = 6.70, 9.28 Hz, H-1'), 3.78 (s, 3H, OCH_3), 4.37 (d 1H, *J* = 6.87 Hz, H-6), 6.17-6.23 (m, 1H, H-3, H-4), 7.23 (d, 1H, *J* = 5.33 Hz, H-2); ^{13}C NMR ($CDCl_3$): δ 13.95 (C-4'), 19.36 (C-3'), 30.34 (C-2'), 32.15 (C-5), 51.78 (O<u>C</u>H_3), 67.08 (C-6), 68.32 (C-1'), 122.86 (C-3), 126.22 (C-1), 133.15 (C-4), 135.46 (C-2), 167.76 (C=O); LR-MS (ES^+): 228.2 $[M+NH_4]^+$.

4-(α-D-Glucose-2',3',4',6'-tetraacetate)-cyclohexa-1,5-dienecarboxylic acid methyl ester 16a and 16b. A solution of compound vinyl sugar **9a** (1.07 g, 2.86 mmol, 2 eq.) and 3-carbomethoxy-2(H)-pyran-2-one **13** (220 mg, 1.43 mmol, 1 eq.) in DCM (1 mL) was heated for 6 days at 60 °C in a sealed tube. The reaction mixture was cooled to room temperature and the solvent was removed under reduced pressure. The crude product was purified by flash chromatography on silica gel (pre-washed with petrol/EtOAc/Et_3N (92:6:12)), eluting with petroleum ether\EtOAc (5:1) to give compound **16a** (374 mg, 32%) as yellow oil and compound **16b** (194 mg, 25%) as a yellow oil.

*Compound **16a*** IR: (thin film) 2951, 2879, 1758, 1644, 1437, 1369, 1221, 1038, 914 cm^{-1}. ^{1}H NMR ($CDCl_3$, 600 MHz): δ 1.97 (s, 3H, CH_3), 1.98 (s, 3H, CH_3), 1.99 (s, 3H, CH_3), 2.03(s, 3H, CH_3), 2.36-2.42 (dt 1H, J = 14.1, 1.7 Hz, H-3), 2.77 (dd, 1H, J = 5.3, 14.6 Hz, H-3), 3.53-3.57 (m, 1H, H-6'), 3.67 (dd, 1H, J = 3.1, 11.0 Hz, H-6'), 3.71 (dt, 1H, J = 3.4, H-5'), 3.77 (s, 3H, OCH_3), 4.40 (dd, 1H, J = 6.9, 14.6 Hz, H-4), 5.04 (t, 1H J = 9.5 Hz, H-2'), 5.13 (t, 1H, J = 9.5 Hz, H-4'), 5.18 (t, 1H, J = 9.6 Hz, H-3'), 5.66 (d, 1H, J = 8.3 Hz, H-1'), 6.14-6.18 (m, 1H, H-1), 6.21-6.24 (m, 1H, H-2), 7.20 (d, 1H, J = 5.5 Hz, H-5a). ^{13}C NMR ($CDCl_3$, 150.9 MHz): δ 20.66 (CH_3), 20.70 (CH_3), 20.78 (CH_3), 20.95 (CH_3), 30.52 (C-6), 51.84 (OCH_3), 61.50 (C-4), 66.67 (C-6'), 67.79 (C-4), 68.74 (C-4'), 70.44 (C-2'), 73.17 (C-3'), 73.74 (C-5'), 91.84 (C-1'), 122.58 (C-1), 125.26 (C-5), 133.52 (C-2), 136.05 (C-5a), 167.64 (C=O), 169.12 (C=O), 169.37 (C=O), 169.47 (C=O), 170.32 (C=O). LRMS (ESI$^+$): 502.1 $[M+NH_4]^+$. HRMS (ESI) calcd for $C_{22}H_{32}O_{12}N$ $[M+NH_4]^+$: 502.1919; Found: 502.1917.

*Compound **16b*** ^{1}H NMR ($CDCl_3$, 600 MHz): δ 2.03 (s, 3H, CH_3), 2.08 (s, 3H, CH_3), 2.08 (s, 3H, CH_3), 2.11(s, 3H, CH_3), 2.36-2.42 (m 1H, J = 1.7, 14.1 H-3), 2.77 (dd, 1H, J = 5.3, 14.6 Hz, H-3), 3.53-3.57 (m, 1H, H-6'), 3.67 (dd, 1H, J = 3.1, 11.0 Hz, H-6'), 3.79 (s, 3H, OCH_3), 4.40 (dd, 1H, J = 6.9, 14.6 Hz, H-4), 5.07 (t, 1H J = 9.5 Hz, H-2'), 5.12 (t, 1H, J = 9.5 Hz, H-4'), 5.24 (t, 1H, J = 9.5 Hz, H-3'), 5.71 (d, 1H, J = 8.4 Hz, H-1'), 6.14-6.18 (m, 1H, H-1), 6.21-6.24 (m, 1H, H-2), 7.24 (d, 1H, J = 5.7 Hz, H-5a). ^{13}C NMR ($CDCl_3$, 150.9 MHz): δ 20.66 (CH_3), 20.70 (CH_3), 20.91 (CH_3), 21.14 (CH_3), 30.61 (C-6), 51.91 (OCH_3), 60.48 (C-4), 66.85 (C-6'), 68.06 (C-4), 68.62 (C-4'), 70.28 (C-2'), 73.14 (C-3'), 74.37 (C-5'), 91.76 (C-1'), 122.58 (C-1), 125.11 (C-5), 133.50 (C-2), 135.99 (C-5a), 167.57 (C=O), 169.04 (C=O), 169.40 (C=O), 170.18 (C=O), 170.69 (C=O). LRMS (ESI$^+$): 502.1 $[M+NH_4]^+$. HRMS (ESI) calcd for $C_{22}H_{32}O_{12}N$ $[M+NH_4]^+$: 502.1919; Found: 502.1917.

Epoxidation of compounds 16a and 16b Compound **16a** (350 mg, 0.72 mmol) was dissolved in DCM (10 mL) and mCPBA (65% w/w, 200 mg, 0.72 mmol) was added. The reaction mixture was stirred at room temperature for 24 h. The reaction was quenched by addition of 1 M $NaHSO_4$ (5 mL) and extracted with dichloromethane (3 x 25 mL). The organic layer was washed with $NaHCO_3$ (15 mL) and dried over $MgSO_4$ and concentrated under reduced pressure. The crude product was purified by flash chromatography on silica gel, eluting with petroleum ether/EtOAc (7:1 then 5:1) to give compounds **17a** (241 mg, 67%) and **17b** (90 mg, 25%)

*Compound **17a***

IR: (thin film) 3022, 2944, 1760, 1721, 1373, 1368, 1260, 1217, 1165, 1073, 849 cm^{-1}. ^{1}H NMR ($CDCl_3$): δ 1.66 (dd, 1H, J = 5.0, 16.0 Hz, H-3), 2.02 (CH_3), 2.02 (CH_3), 2.03 (CH_3), 2.03 (CH_3), 2.55 (dm, 1H, J = 16 Hz, H-3), 3.43 (t, 1H, J = 4.1 Hz, H-1), 3.47 (dd, 1H, J = 4.8, 11.3 Hz, H-6'), 3.64 (m, 1H, H-2), 3.60 (dd, 1H, J = 2.8, 6.4 Hz, H-6'), 3.72 (ddd, 1H, J = 2.8, 5.0, 10.0 Hz, H-5'), 3.78 (s, 3H, OCH_3), 4.06 (m, 1H, H-4), 5.07-5.10 (m, 2H, H-2', H-4'), 5.21 (t, 1H, J = 9.5 Hz, H-3'), 5.65 (dd, 1H, J = 8.4, 11.3 Hz, H-1'), 7.41 (dd, 1H, J = 1.4, 4.3 Hz, H-5a). ^{13}C NMR ($CDCl_3$): δ 20.7 (CH_3), 20.81 (CH_3), 20.94 (CH_3), 21.18 (CH_3), 26.27 (C-3), 45.36 (C-1), 52.22 (OCH_3), 55.70 (C-2), 67.84 (C-6'), 69.02 (C-4), 70.23 (C-4'), 72.87 (C-3'), 74.10 (C-5'), 80.55 (C-2'), 91.78 (C-1'),132.29 (C-5), 140.25 (C-5a), 165.84 (C=O), 169.24 (C=O),169.48 (C=O), 170.01 (C=O), 170.24 (C=O).

*Compound **17b***

^{1}H NMR ($CDCl_3$): δ 2.02 (CH_3), 2.02 (CH_3), 2.03 (CH_3), 2.03 (CH_3), 2.21 (ddd, 1H, J = 4.0, 5.7, 15.5 Hz, H-3), 2.40 (ddd, 1H, J = 2.2, 6.2, 15.5 Hz, H-3), 3.33 (t, 1H, J = 3.9 Hz, H-1), 3.47 (dd, 1H, J = 4.8, 11.3 Hz, H-6'), 3.60 (m, 2H, H-2 and H-6'), 3.72 (dm, 1H, J = 10.0 Hz, H-5'), 3.78 (s, 3H, OCH_3), 4.56 (m, 1H, H-4), 5.07-5.10 (m, 2H, H-2', H-4'), 5.21 (t, 1H, J = 9.5 Hz, H-3'), 5.65 (dd, 1H, J = 8.4, 11.3 Hz, H-1'), 7.41 (dd, 1H, J = 1.4, 4.3 Hz, H-5a). ^{13}C NMR ($CDCl_3$): δ 20.7 (CH_3), 20.81 (CH_3), 20.94 (CH_3), 21.18 (CH_3), 26.27 (C-3), 45.36 (C-1), 52.22 (OCH_3), 55.70 (C-2), 67.84 (C-6'), 69.02 (C-4), 70.23 (C-4'), 72.87 (C-3'), 73.10 (C-5'), 82.55 (C-2'), 105.80 (C-1'),132.29 (C-5), 140.25 (C-5a), 165.84 (C=O), 169.24 (C=O),169.48 (C=O), 170.01 (C=O), 170.24 (C=O). LRMS (ESI$^+$): 501.1 $[M+H]^+$. HRMS (ESI) calcd for $C_{22}H_{29}O_{13}$ $[M+H]^+$: 501.4579; Found: 501.4584.
Using similar procedure as above, compound **16b** (150 mg, 0.31 mmol) give compounds **17c** (36 mg, 23%) and **17d** (87 mg, 56%)

Conversion of compounds 17a-17d to compounds 18a - 18d

A solution of compound **17a** (220 mg, 0.44 mmol) in DME/EtOH/H_2O (2:1:1, 8 mL) was cooled to 0 °C. Sodium azide (280 mg, 4.4 mmol) was added followed by NH_4Cl (235 mg, 4.4 mmol). The reaction mixture was stirred at 0 ^oC for 1 h and then stirred at room temperature for 24 h. The reaction mixture was concentrated under reduced pressure, diluted with water (15 mL) and extracted with EtOAc (4 x 50 mL). The combined organic layers were washed with brine, dried over $MgSO_4$ and concentrated under reduced pressure. The crude product was was suspended in toluene (5 mL) and filtered to remove any insoluable material. The solution was cooled to -78 ^oC under a dry nitrogen atmosphere and $^iBu_2Al$-H (4.5 mL, of a 1 M solution in toluene, 10 eq.) was added dropwise. After 5 hours the reaction mixture was quenched at -78 ^oC by addition of a 1 M solution of potassium sodium tartarate (5 mL) and then EtOAc (15 mL). The reaction mixture was warmed to room temperature and stirred for 30 mins. The organic layer was separated and the aqueous phase was extracted with EtOAc (4 x 50 mL). The combined organic extracts were dried over $MgSO_4$ and concentrated under reduced pressure. The crude product was purified by flash chromatography on silica gel, eluting with petroleum ether/EtOAc (5:1) to give the compound **18a** (195 mg, 86%) as a pale yellow oil. Compounds **18b**, **18c** and **18d** were prepared similarly.

*Compound **18a***

^{1}H NMR ($CDCl_3$): δ 1.71 (dt, 1H, *J* = 12.7, 10.1 Hz, H-3), 2.02 (CH_3), 2.02 (CH_3), 2.03 (CH_3), 2.03 (CH_3), 2.51 (ddd, 1H, *J* = 3.6, 5.5, 12.7 Hz, H-3), 3.37 (dd, 1H, *J* = 4.8, 11.3 Hz, H-6'), 3.62 (m, 2H, H-2 and H-6'), 3.72 (ddd, 1H, *J* = 2.8, 5.0, 10.0 Hz, H-5'), 3.92-3.94 (m, 1H, H-1), 4.04-4.10 (m, 2H, H-6), 4.27-4.29 (m, 1H, H-4), 5.07-5.10 (m, 2H, H-2', H-4'), 5.21 (t, 1H, *J* = 9.5 Hz, H-3'), 5.57 (m, 1H, H-5a), 5.65 (dd, 1H, *J* = 8.4, 11.3 Hz, H-1'), 7.41 (dd, 1H, *J* = 1.4, 4.3 Hz, H-5a). ^{13}C NMR ($CDCl_3$): δ 20.7 (CH_3), 20.81 (CH_3), 20.94 (CH_3), 21.18 (CH_3), 37.53, (C-3), 63.75 (C-6), 65.93 (C-1), 67.84 (C-6'), 70.23 (C-4'), 71.71 (C-2), 72.30 (C-4), 72.87 (C-3'), 74.10 (C-5'), 80.55 (C-2'), 91.78 (C-1'), 124.99 (C-5a), 141.00 (C-5). 169.24 (C=O), 169.48 (C=O), 170.01 (C=O), 170.24 (C=O). HRMS (ESI) calcd for $C_{21}H_{26}O_{11}$ $[M-H_2O,-HN_3]^+$: 454.1475; Found: 454.1478.

Assessment of heparanase inhibition.

Genway heparanase degrading enzyme assay kit (cat 40-831-160016) was used and the procedure was modified as follows. In a 96-well plate, 40 μL of a solution of inhibitor at different concentrations (and PBS as control), 10 μL of a recombinant solution of human heparanase (Apollo Scientific, Cheshire UK) prepared from a stock solution of 10 ng/mL, and 50 μL of biotinylated heparan sulfate were mixed and the plate was gently agitated on a shaker for 1 min. The mixture was transferred into CBD-FGF-immobilised 96-well microtitre plate within 30 sec and incubated at 37 °C for 15 min. The reaction solution was removed by aspiration and 100 μL PBS containing 0.1% Tween20 was added. The microtitre plate was gently agitated on a shaker for 1 min, and the solution was removed. The washing process was repeated twice more. 100 μL of avidin POD conjugate solution was added to each well and incubated at 37°C for 30 min. Then, 100 μL of the POD substrates were added to each well, and the colour was allowed to develop at room temperature. After exactly 15 min, 100 μL of 0.5M sulphuric acid was added to each well to stop the reaction. The absorbance of each well was measured at 450 nm. To ensure reproducible results, absorbance should be measured within 5 min. The IC_{50} values were calculated as the conc of each inhibitor required to decrease the maximum absorbance by 50% (Figure 1). Data were analysed with SigmaPlot v8.0.

Molecular modelling.

Alignment of heparanase (sequence from Uniprot accession number Q9Y251) and *Acidobacterium capsulatum* strain ATCC 51196 (sequence from Uniprot accession number C1F2K5) were performed using MOE sequence and structural alignment tool and blosum62 substitution matrix (Figure 2). The two sequences are 18% (24% for the crystallised segment) identical and 31% (38% over crystallised segment) similar. Three-dimensional model building was performed using the MOE homology programme using Amber 99 forcefield. The scoring method was GB/VI. Initially, a database of 25 structures that were each individually refined to an rms gradient of 0.5 Å was generated. The stereochemical quality of the models was checked by using Ramachandran plot analysis and structural analysis.
