# Supplementary figures and images for: Synthesis of a Pseudo-Disaccharide Library and Its Application to the Characterisation of the Heparanase Catalytic Site
Source: PLoS One. 2013 Nov 18;8(11):e82111. doi: 10.1371/journal.pone.0082111 (PMC3832595; doi:10.1371/journal.pone.0082111)

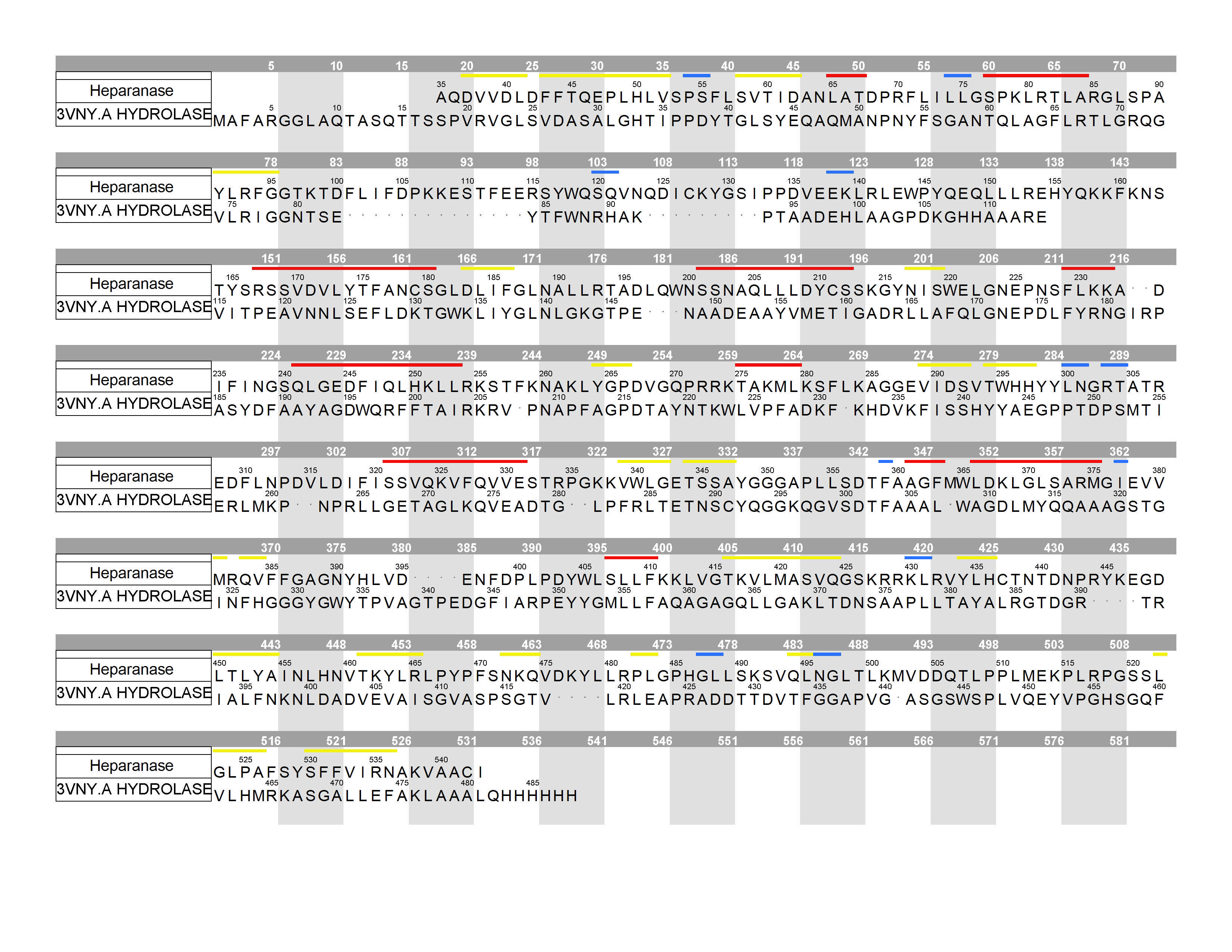

Supplement: File S3 — Alignment of heparanase (sequence from Uniprot accession number Q9Y251) and β-glucuronidase from Acidobacterium capsulatum strain ATCC 51196 (sequence from Uniprot accession number C1F2K5). Predicted folds are shown (red is an α-helix, yellow is β-sheet and blue is a turn). (TIF) [file pone.0082111.s003.tif]
